# Allelopathic Interactions between the Opportunistic Species *Ulva prolifera* and the Native Macroalga *Gracilaria lichvoides*

**DOI:** 10.1371/journal.pone.0033648

**Published:** 2012-04-09

**Authors:** Dong Xu, Zhengquan Gao, Xiaowen Zhang, Xiao Fan, Yitao Wang, Demao Li, Wei Wang, Zhimeng Zhuang, Naihao Ye

**Affiliations:** 1 Yellow Sea Fisheries Research Institute, Chinese Academy of Fishery Sciences, Qingdao, China; 2 School of Life Sciences, Shandong University of Technology, Zibo, China; 3 College of Animal Science and Technology, Qingdao Agricultural University, Qingdao, China; 4 Tianjin Key Laboratory for Industrial Biological Systems and Bioproce Engineering, Tianjin Institute of Industrial Biotechnology, Chinese Academy of Sciences, Tianjin, China; University of Canterbury, New Zealand

## Abstract

Allelopathy, one type of direct plant competition, can be a potent mechanism through which plant communities are structured. The aim of this study was to determine whether allelopathic interactions occur between the opportunistic green tide-forming species *Ulva prolifera* and the native macroalga *Gracilaria lichvoides*, both of which were collected from the coastline of East China sea. In laboratory experiments, the presence of *G. lichvoides* at 1.25 g wet weight L^−1^ significantly inhibited growth and photosynthesis of *U. prolifera* at concentrations of 1.25, 2.50, and 3.75 g wet weight L^−1^ (p<0.05) in both semi-continuous co-culture assays and in co-culture assays without nutrient supplementation. In contrast, although *U. prolifera* had a density effect on *G. lichvoides*, the differences among treatments were not significant (p>0.05). Culture medium experiments further confirmed that some allelochemicals may be released by both of the tested macroalgae, and these could account for the observed physiological inhibition of growth and photosynthesis. Moreover, the native macroalgae *G. lichvoides* was a stronger competitor than the opportunistic species *U. prolifera*. Collectively, the results of the present study represent a significant advance in exploring ecological questions about the effects of green tide blooms on the macroalgal community.

## Introduction

The introduction and spread of exotic species into the marine environment is considered to be a major threat to marine ecosystems, with potentially dramatic effects on biological diversity, productivity, habitat structure, and fisheries [1]. Beginning in the 1990 s, a vast increase in the worldwide spread of nonindigenous organisms has occurred, due mainly to dispersal via human-mediated transport [2], [3], [4]. One of the most representative examples linked with anthropogenic activities is an expansive “green tide” caused by the proliferation of green macroalgae belonging to the genus *Ulva*. Green tides have occurred almost in many enclosed marine water bodies, including in Europe, Australia, New Zealand, Hong Kong, the Philippines, Indonesia, India, Egypt, China, South Africa and Central America [5]. In China between May and July 2008, prior to the Olympic sailing competition, the large scale blooms of *Ulva prolifera* caused the world's largest green tide [6]; it consisted of more than 1 million tons of drifting biomass and covered an area of 13,000–30,000 km^2^ [7], [8].

While the occurrence and spread of *U. prolifera* have been well documented, the mechanism by which it invades a community and its impact on native communities has received little attention. The few studies conducted to date have shown that in addition to having negative effects on tourism, large algal mats can have deleterious ecological effects. These effects include uncoupling of the biogeochemical cycles in sediments from those in the water column [9], a negative impact on seagrass beds due to shading, disruption of feeding by wading birds [10], development of a lethal environment due to oxygen deficiency [11], and a shift from a high-diversity mixture to low-diversity assemblages of fast-growing annual algae [12].

Invasive macroalgae can impact native species through competition for different resources such as light, space, or nutrients [13], [14]; via modification of abiotic stress [15]; and by chemical means, such as allelopathy [16], [17]. Marine seaweeds produce a wide variety of secondary metabolites such as terpenes, sterols, polyphenols, and acetogenins [18]. This phenomenon of interactions among algal species has been called allelopathy. Several recent studies revealed that some of these compounds function as chemical defenses that are able to deter a broad range of natural enemies, including competitors, epiphytes, pathogenic bacteria, and herbivores [19], [20].

The bulk of research on allelopathy has focused on macroalga-microalga interactions, especially in the red tide-inhibition realm. There is now a considerable amount known about allelopathic effects of green macroalgal such as *Ulva fasciata*, *Ulva lactuca*, *Ulva pertusa*, and *Ulva linza* on the harmful microalgae *Prorocentrum micans, Prorocentrum donghaiense, Heterosigma akashiwo*, *Alexandrium tamarense*, and *Chaetoceros gracile* [17], [21], [22]. Additionally, microalga-microalga allelopathic interactions were also found between *P. micans* and *Skeletonema costatum* or *Karenia mikimotoi* [23]. However, much less is known about macroalga-macroalga allelopathic interactions. Our previous study showed that the green tide-forming macroalga *U. linza* could release allelochemicals that could inhibit the growth of the red macroalga *Gracilaria lemaneiformis* (unpublished data).

Like *U. linza*, *U. prolifera* is a dominant species responsible for forming green tides. However, Liu *et al.* (2010) [24] and Zhang *et al.* (2011) [25] reported that the dominant *Ulva* strain of the 2008 green algal bloom in the Yellow Sea was not detected in the coastal waters of Qingdao in the following winter. Compared to *U. linza*, much less is known about the allelopathic ecology of *U. prolifera*, and to date no studies have assessed what happens to the structure and biodiversity of a community when this opportunistic species invades it. In fact, few studies have addressed allelopathic interactions in the marine environment and the function of secondary metabolites as defenses against pathogens or other competing plants. Thus, in this study we examined the allelopathic interactions between the opportunistic species *U. prolifera* and the native macroalga *Gracilaria lichvoides*, both of which were collected from the coastline of East China Sea. We performed a series of laboratory experiments under controlled conditions in which we co-cultured the two species and also cultured them separately (i.e., mono-cultures). Physiological parameters such as algal growth, algal photosynthesis, nutrient assimilation, and changes of pH in the culture medium were examined to assess potential allelopathic effects between the tested macroalgal species.

## Materials and Methods

### Sampling and Culture Conditions

Floating specimens of *U. prolifera* and *G. lichvoides* were collected from the coastline of East China sea, in May 2011. In the laboratory, the intact samples were washed several times with sterile seawater, sterilized with 1% sodium hypochlorite for 2 min, and then rinsed with autoclaved seawater. Both *U. prolifera* and *G. lichvoides* were pre-cultured aseptically in f/2 medium in an incubator without N or P supplement for 48 h before running experiments. The temperature was maintained at 15°C. Illumination was provided by cool-white flouorescent lamps at 100 µmol photons m^−2^ s^−1^ and on a 12∶12 h light: dark cycle. All cultures were shaken manually twice at the same time every day. The pH and salinity of the seawater used for experiment were 8.0 and 30 ppt, respectively.

### Effects of Fresh Thalli of *U. prolifera*/*G. lichvoides* on *G. lichvoides/U. prolifera*

To determine the allelopathic interactions between the fresh thalli of *G. lichvoides* and *U. prolifera*, in the batch culture experiment, *G. lichvoides* (1.25 g wet weight L^−1^) was co-cultured with three different density of *U. prolifera* (1.25, 2.50, and 3.75 g wet weight L^−1^). The experiments were conducted in 500 ml flasks containing 400 ml of culture medium with 882 µmol L^−1^ NaNO_3_ and 32 µmol L^−1^ KH_2_PO_4_ at 15°C and 100 µmol photons m^−2^ s^−1^. During the period of experiments, nutrients were not added into any flask to supply the decreasing of the nutrients. In addition, a serial of semi-continuous experiments were also conducted by regularly adding nutrients to 882 µmol L^−1^ NaNO_3_ and 32 µmol L^−1^ KH_2_PO_4_ every 24 h, while the culture conditions were the same as described above. *G. lichvoides* and *U. prolifera* were individually cultured (monocultured) as controls. All experiments in this study were conducted at in triplicate, and aseptic techniques were used in all experimental steps. Flasks were also monitored for pH levels during the experiments. Measurements were taken using a pH probe equipped with an electrode (Thermo Scientific Orion Star SeriesTM Benchtop pH meter; ±0.01 unit; calibrated prior each use with NIST traceable standards). These experiments lasted for 96 h. The growth of macroalgae *U. prolifera* and *G. lichvoides* was estimated by monitoring changes in algal wet weight at 0 h, 48 h, and 96 h.

### Effects of Culture Filtrate of *U. prolifera*/*G. lichvoides* on *G. lichvoides/U. prolifera*

Macroalgal culture medium was prepared by separately culturing *G. lichvoides* and *U. prolifera* in sterilized seawater at a concentration of 10 g wet weight L^−1^ for 48 h without nutrient enrichment. Thereafter, the macroalgal thalli were removed and the macroalga-free culture medium was filtered through 0.45 µm acetate cellulose filters and diluted 2 and 4 times with sterilized seawater. The three gradient concentrations of culture filtrate of *G. lichvoides* or *U. prolifera* were used for experiments to study the effects of culture filtrates on fresh algal thalli of *U. prolifera* or *G. lichvoides* at a concentration of 1.25 g wet weight L^−1^. The media containing culture filtrates were resupplied with nutrients every 24 h, and the pH was adjusted to 8.0 by 2 mol L^−1^ HCl every day. The culture system was kept at 15°C with a light intensity of 100 µmol photons m^−2^ s^−1^ and a 12∶12 h light: dark cycle. The experiments lasted for 96 h, and the growth of macroalgae *U. prolifera* and *G. lichvoides* was estimated by monitoring changes in algal wet weight at 0 h, 48 h, and 96 h.

### Nutrient Analysis

During the experiments, water samples (5 ml) in batch culture were collected daily, filtered immediately through acetate cellulose filters, and frozen in polyethylene flasks for storage until analysis. Concentrations of nitrate (NO_3_–N) and phosphas (PO_4_–P) were analyzed photometrically using an AutoAnalyzer (BRAN and LUEBBE AA3, Germany). Nutrient uptake rates were calculated as: *NUR* = (*C_0_*−*C_t_*) *V*/*DW*/*t*, where *NUR* is the nutrient uptake rate (µmol of nutrient g FW wt^−1^ h^−1^); *C_0_* and *C_t_* are the nutrient concentrations (µmol L^−1^) at the beginning and the end of the experiment, respectively; *V* is the volume of water (L); *FW* is the algal fresh weight (g), and *t* is the time interval (h).

### Photosynthesis Measurement

Simultaneously, the effective PSII quantum yield [*Y(II)*] of fresh algal thalli was measured using the pulse–amplitude modulated method on a Dual-PAM-100 (Walz, Effeltrich, Germ any) connected to a PC running WinControl software and calculated as follows: *Y(II)* = (*F*_m_′−*F*_t_)/*F*_m_′. The real-time fluorescence yield *F*_t_ is obtained by averaging the fluorescence readings within 0.2 s and the maximum fluorescence yield (*F*_m_′) was detected when the samples were illuminated by actinic light of 100 µmol photons m^−2^ s^−1^.

### Statistical Analysis

The significance of variance between treatments and the control or among treatments was tested using a one-way ANOVA or a multiple comparison test. All tests were run using the software SPSS 17.0. The significance level was set at 0.05 for all tests unless otherwise stated.

## Results

### Effects of fresh thalli of *U. prolifera*/*G. lichvoides* on *G. lichvoides*/*U. prolifera*

**Figure 1:**
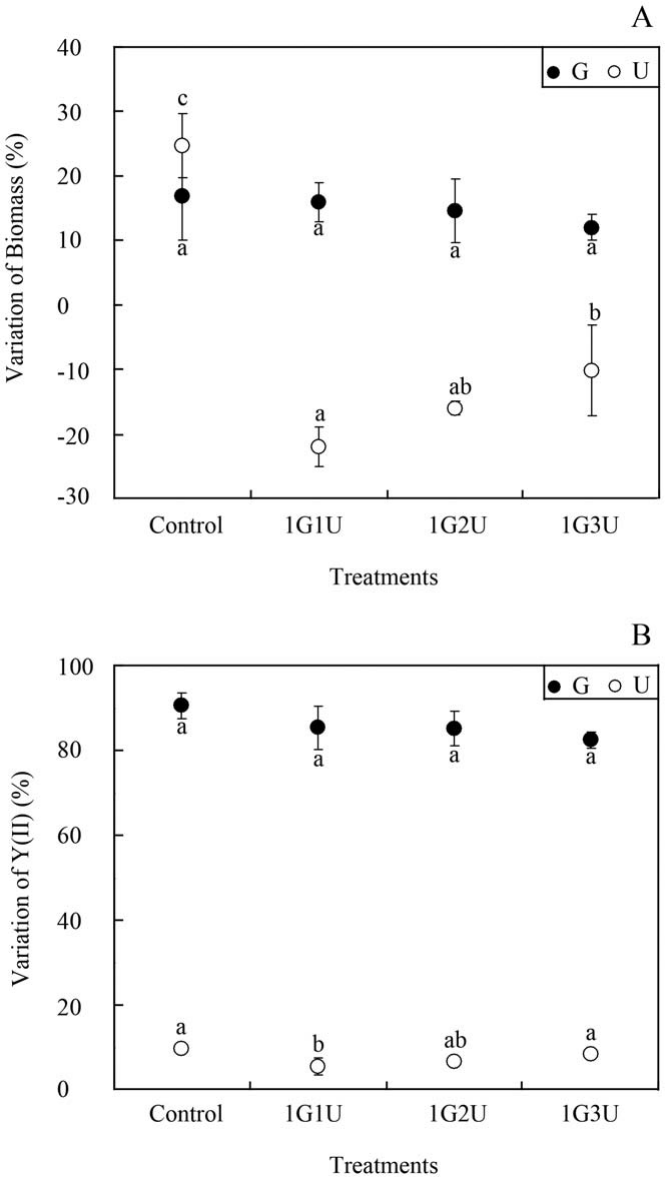
Interactions between *U. prolifera* and *G. lichvoides* in fresh thalli batch co-culture experiment. A) Growth-inhibition effects, B) Photosynthetic effects. Values (means ± SD) in bars that have the same letter are not significantly different (*p*>0.05).

In the batch co-culture experiments without nutrient supplement, different quantities of *U. prolifera* had no apparent effects on the growth (df = 3, F = 0.467, p = 0.718) or photosynthesis (df = 3, F = 2.191, p = 0.232) of *G. lichvoides* compared to the control (Fig. 1A and B). After 96 h of incubation, the biomass and *Y(II)* of the mono-cultured *G. lichvoides* increased by 17.0±7.1% and 90.6±2.5%, respectively. In the three co-culture systems containing 1.25, 2.50, and 3.75 g wet weight L^−1^ of *U. prolifera*, *G. lichvoides* biomass increased by 16.0±2.8%, 14.7±4.6%, and 12.0±2.1%, respectively, and *Y(II)* increased by 85.4±4.5%, 84.4±4.2%, and 82.5±2.0%, respectively. In contrast, the presence of *G. lichvoides* at a concentration of 1.25 g wet weight L^−1^ had a dramatic negative effect on growth of *U. prolifera* at concentrations of 1.25, 2.50, and 3.75 g wet weight L^−1^ (growth declined by 22.0±2.8%, 16.0±1.0%, and 10.2±7.0%, respectively) (df = 3, F = 4.968, p = 0.046). Moreover, although the *Y(II)* of *U. prolifera* increased by 5.4±1.6%, 6.7±0.6%, and 8.4±0.7%, the effects of *G. lichvoides* on photosynthesis of *U. prolifera* were not significant compared to the control treatment (df = 3, F = 4.363, p = 0.073).

**Figure 2:**
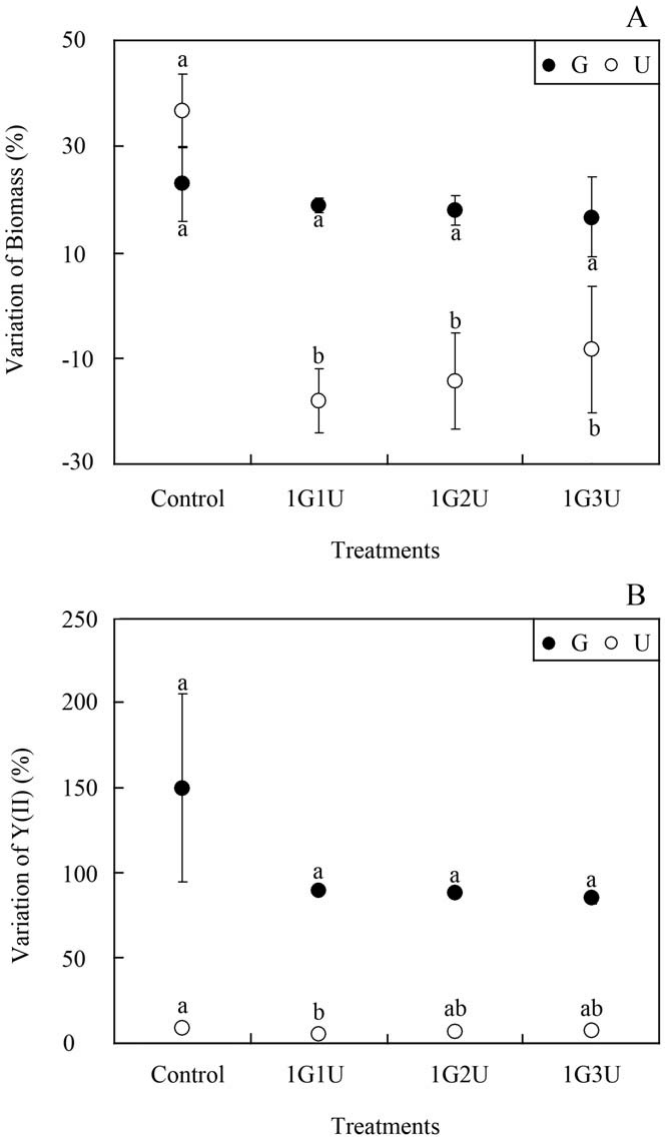
Interactions between the fresh thalli of *U. prolifera* and *G. lichvoides* in semi-continuous cultivation. A) Growth-inhibition effects, B) Photosynthetic effects. Values (means ± SD) in bars that have the same letter are not significantly different (*p*>0.05).

*G. lichvoides* also grew well both in monoculture and in co-cultures with *U. prolifera* (Fig. 2A and B) in semi-continuous cultivation conditions. By the end of the experiment, the biomass of mono-cultured *G. lichvoides* increased by 23.0±7.1% and that in the three co-culture treatments increased by 19.0±1.4%, 18.0±2.8%, and 16.7±7.6%, respectively. Similarly, *Y(II)* values in the co-culture treatments increased by 90.1±1.6%, 88.0±2.4%, and 85.4±3.4%, respectively, although all of these values were lower than that of the control, which increased by 150.2±55.2%. However, one-way ANOVA indicted that these differences were not significant (df = 3, F = 2.550, p = 0.194). In contrast, *G. lichvoides* had density-dependent effects on growth and photosynthesis of *U. prolifera*. After incubation for 96 h, the biomass of *U. prolifera* declined significantly by 18.0±5.7%, 14.3±9.5%, and 8.3±11.8%, respectively, compared to the control, which increased by 36.7±6.7% (df = 3, F = 17.157, p = 0.005). However, *Y(II)* of *U. prolifera* did not change significantly (df = 3, F = 1.619, p = 0.281).

### Nutrient changes in fresh thalli co-culture

**Figure 3:**
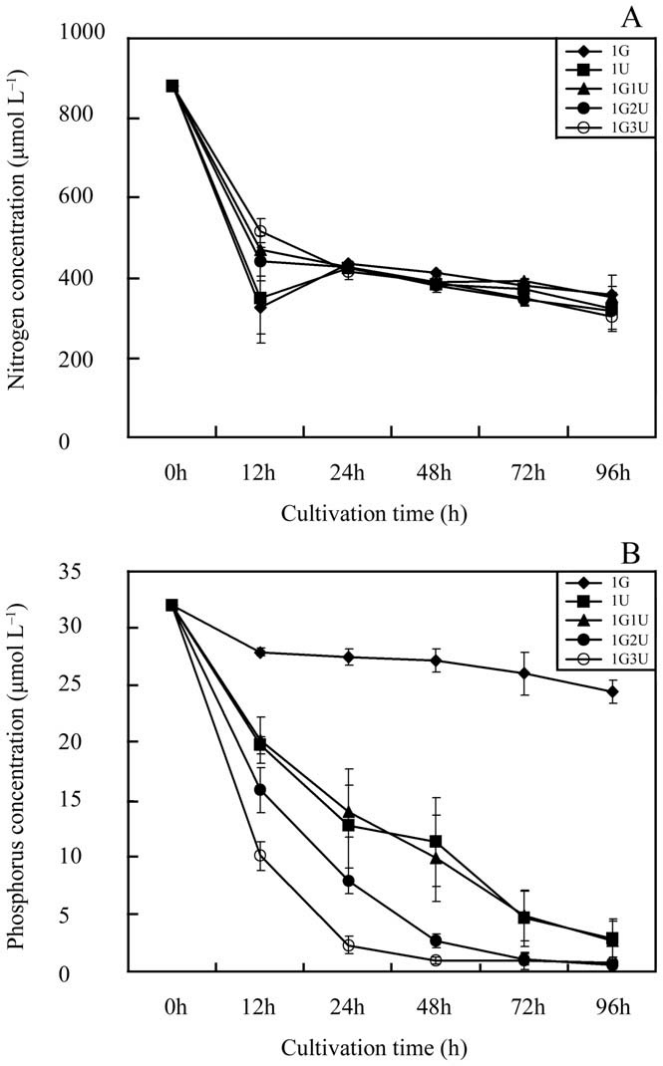
Variations of nutrient concentration with culture time in fresh thalli batch co-culture experiment. A) Changes in nitrate concentrations, B) Changes in phosphorus concentrations.

Fig. 3 shows changes in nutrient concentrations with culture time in the fresh thalli batch culture systems. The NO_3_–N concentration in the monoculture of *U. prolifera* decreased more quickly (from 882 to 325.0±53.1 µm L^−1^) than that in the monoculture of *G. lichvoides* (882 to 359.4±47.1 µm L^−1^), except for the first 12 h. The NO_3_–N concentration in the monoculture of *U. prolifera* and in the monoculture of *G. lichvoides* was significantly correlated with the concentration in the co-culture systems; this relationship illustrates that NO_3_–N in the co-culture assays was absorbed jointly by *G. lichvoides* and *U. prolifera* (Fig. 3A). During the period the 96 h, the average N uptake rate of *G. lichvoides* (4.5±0.4 µmol N g^−1^ FW h^−1^) in monoculture experiment was lower than that of *U. prolifera* (4.8±0.4 µmol N g^−1^ FW h^−1^), but the difference was not significant (df = 1, F = 0.704, p = 0.449).

The concentration of PO_4_–P in the monoculture of *U. prolifera* was significantly correlated with that in the co-culture of *G. lichvoides* with *U. prolifera*. Moreover, the PO_4_–P concentration in the co-culture system declined to much lower levels (from 32 to 2.6±1.8, 0.5±0.3, and 0.7±0.5 µm L^−1^, respectively) compared with that in the *G. lichvoides* monoculture (24 µm L^−1^) after 96 h. These results indicate that the PO_4_–P was mainly absorbed by *U. prolifera* in all co-culture assays (Fig. 3B). Moreover, the average P uptake rate of *G. lichvoides* (0.08±0.01 µmol P g^−1^ FW h^−1^) in monoculture experiment was dramatically higher than that of *U. prolifera* (0.25±0.01 µmol P g^−1^ FW h^−1^) (df = 1, F = 340.099, p = 0.000).

### pH changes in fresh thalli co-culture

The pH of the culture medium used in both the monoculture and co-culture systems initially was 8.0. Over time, the pH values in all treatments increased slightly (no more than 1.12) in both the semi-continuous assays and in the co-culture assays without nutrient supplementation (Table 1). A concentration-dependent relationship was observed between the initial concentration of fresh thalli and pH values measured after 96 h of incubation.

**Table 1:** Changes of pH values with culture time in the fresh thalli co-culture.

Treatment	In no nutrients added assays		In semi-continuous assays	
Time(h)	48 h	96 h	48 h	96 h
1G	8.01	8.14	8.02	8.11
1U	8.54	8.69	8.51	8.63
1G1U	8.56	8.74	8.53	8.69
1G2U	8.85	9.02	8.60	8.78
1G3U	8.93	9.12	8.80	8.91

### Effects of culture filtrates of *U. prolifera*/*G. lichvoides* on *G. lichvoides*/*U. prolifera*

**Figure 4:**
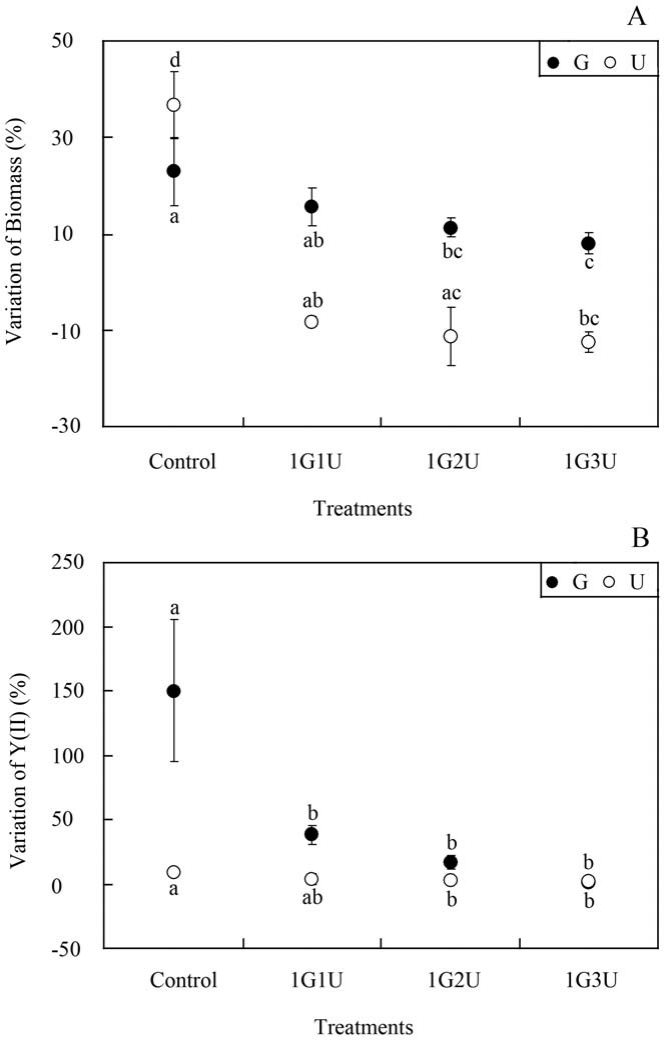
Effects of macroalgal culture filtrates in semi-continuous cultivation. A) Growth-inhibition effects, B) Photosynthetic effects. Values (means ± SD) in bars that have the same letter are not significantly different (*p*>0.05).

Fig. 4 shows results of the experiments in which *U. prolifera* or *G. lichvoides* was cultured with macroalgal culture filtrates of *G. lichvoides* or *U. prolifera*, respectively. The *G. lichvoides* culture filtrate dramatically inhibited growth (df = 3, F = 55.759, p = 0.001) and photosynthesis (df = 3, F = 2.923, p = 0.139) of *U. prolifera* in comparison to the control (Fig. 4). After 96 h of incubation, the biomass of *U. prolifera* decreased by 8.1±1.2%, 11.3±5.7%, and 12.4±1.5%, respectively, when treated with 4, 2, and 1 times diluted culture filtrate of *G. lichvoides*, whereas the biomass in the control increased by 36.7±6.7%. Additionally, although the *Y(II)* values of *U. prolifera* increased by 3.8±4.6%, 3.1±2.7%, and 1.9±1.7%, the 2 and 1 times diluted culture filtrates of *G. lichvoides* had significant effects on photosynthesis of *U. prolifera*. Moreover, the *U. prolifera* culture filtrate also caused significant inhibition of growth (df = 3, F = 7.239, p = 0.015) and photosynthesis (df = 3, F = 11.627, p = 0.019) of *G. lichvoides* compared with those of the control. In the monoculture, the biomass and *Y(II)* value of *G. lichvoides* increased by 23.0±7.1% and 150.2±55.2%, respectively. When treated with 4, 2, and 1 times diluted culture filtrate of *U. prolifera*, the biomass and *Y(II)* of *G. lichvoides* increased by only 15.7±3.6%, 11.3±2.1%, and 8.1±2.3% and 38.5±7.4%, 16.9±5.1%, and 1.3±0.1%, respectively.

## Discussion

The direct competitive effects of exotic plants on natives are among the leading causes of plant extinctions worldwide [26]. It's known that there are multiple mechanisms such as resource competition [13], environmental factors [16] and/or negative allelopathy [18], that may account for the negative interactions. Among direct competitive interactions, resource competition is frequently credited as being the principal competitive mechanism that affects plant success. Huo *et al.* (2011) [13] indicated that the growth of *K. mikimotoi* was suppressed by *Gracilaria verrucosa* mainly through competition for nutrients, especially nitrogen. Previous studies have reported that the green macroalgae *Ulva spp.*, which have a high surface area/volume ratio, exhibit high rates of nutrient uptake. But many of these algae are often limited in their ability to concentrate and store nitrogen internally, and are, therefore dependent on a constant high level of nitrogen in the medium [27]. The red macroalge *Gracilaria spp*., on the other hand, have opposite qualities in that they show a high capability of storing nitrogen (partly as phycoerythrin), and require only pulse fertilization [28]. In the present study, high-level nutrient assimilation clearly occurred in the fresh thalli co-culture experiments with three *U. prolifera* concentrations (Fig. 3) without nutrient supplementation. And the *U. prolifera* appeared higher nutrient uptake than *G. lichvoides*. The NO_3_–N in the co-culture assays was absorbed jointly by *G. lichvoides* and *U. prolifera*, but PO_4_–P was mainly absorbed by *U. prolifera*. Correspondingly, *U. prolifera* biomass dramatically declined by 22.0±2.8%, 16.0±1.0%, and 10.2±6.7%, respectively, after incubation for 96 h (Fig. 1). Thus, it seemed that resource competition likely accounted for the observed growth suppression. However, in the semi-continuous assays (Fig. 2), in which nutrients were added every 24 h, *G. lichvoides* also had density-dependent effects on the growth of *U. prolifera*. After incubation for 96 h, the biomass of *U. prolifera* declined by 18.0±5.7%, 14.3±9.5%, and 8.3±1.7%, whereas that in the control increased by 37%. Therefore, nutrient limitation could be excluded as the cause of the observed negative effects.

Light competition was another mechanism that account for the species interactions. Tait and Schiel (2011) [29] indicated that the light intensity played an important role in productivity of canopy-forming macroalgae and their sub-canopy assemblages. At high cover, *Sargassum muticum* excludes native species and reduces richness through light competition by shading smaller, understory macroalgae [30]. Inversely, Svirski *et al.* (1993) [31] found that the growth inhibition of *Gracilaria spp.*, when cultured in the presence of *Ulva cf. lactuca*, was not due to shading or nutrient depletion, but seemed to be caused by competition for inorganic carbon or some type of allelopathy. In the present study, the fresh algae was incubated in 500 ml flasks containing 400 ml of culture medium, and the space was big enough for the sample to growth. Meanwhile, the experiment was conducted in an illuminated incubator at 100 µmol photons m^−2^ s^−1^ and the algae can get the light from all directions. Additionally, all cultures were shaken manually twice every day and the samples could change their positions in the culture medium. Based on mentioned above, it makes light limit unlikely for growth.

Allelopathy, which is one type of direct plant competition, can play an important role in ecosystem structure and plant diversity [18]. Although the importance of allelopathy as a mechanism of competition is gaining prominence in terrestrial ecological research, the importance of allelopathy in aquatic ecosystems has received less attention, especially among macroalgae [32]. A recognized effect of growing macroalgae in culture is that they may increase the pH of the culture medium, making it unsuitable for the growth of microalgae in co-culture [33], [34]. In our experiments, the pH value of the culture medium was measured at the beginning and the end of the experiment, which increased (no more than 1.12) both in batch co-culture assays and in the semi-continuous assays. The pH changes may result in the growth inhibition of *G. lichvoides* or *U. prolifera*. However, in the culture filtrate experiments (Fig. 4), in which pH was adjusted to 8.0, the algal growth was also dramatically inhibited. Consequently, the elevated pH values may not the reason cause growth inhibition.

Because exotic plants are the major cause of declining plant diversity and abundance, determining the mechanisms through which exotic plants are able to become invasive could assist in the control and management of these species. It also could provide insight into how plant species interact and how plant communities are organized [26]. Based on the analysis above, neither nutrient and light limitation nor elevated pH was responsible for the observed effects in co-culture systems. In the culture filtrate assays, in which nutrient and pH changes were excluded, significant inhibition of growth and *Y(II)* was found in both experiments (*G. lichvoides* filtrate added to *U. prolifera* culture or *U. prolifera* filtrate added to *G. lichvoides* culture) (Fig. 4). These results indicate that allelochemical compounds may have been released by both of the tested algae. Moreover, the culture filtrate of *G. lichvoides* had a stronger ability to inhibit *U. prolifera* compared to the effect of the culture filtrate of *U. prolifera* on *G. lichvoides*. Collectively, these results provide a new insight about this macroalga-macroalga relationship: Although *U. prolifera* is the causative species of the world's largest green tide and its blooms have major ecological and economic impacts, the presence of a stable native algal canopy of *G. lichvoides* may inhibit its expansion.

### Allelopathic effects of green tide blooms on the native community

Although green tides are widespread and invade many macroalgal ecosystems, they have been largely neglected in studies of the maintenance of biodiversity. The effects of green tide blooms are varied and have been summarized by Fletcher (1996) [35] and Raffaelli *et al.* (1998) [10]. However, our results suggest a more complex picture that involves a chemical-mediated system. Our findings illustrate that the native macroalgae *G. lichvoides* had strong allelopathic effects on the opportunistic species *U. prolifera* when the co-culture concentration of *G. lichvoides* was one-third times higher than that of *U. prolifera*. This may explain why the dominant *U. prolifera* strain of the bloom was absent in all the water-derived cultures during the sampling period [24], [25].

The effects of the introduction and spread of exotic species on community richness can be positive [36], negative [37], or neutral [38]. In addition, the impacts of exotic species are often species specific and context dependent. For example, Valentine and Johnson (2003) [39] reported that disturbance that reduced cover of the native algal canopy was critical in the establishment of *Undaria pinnatifida*, whereas the presence of a stable native algal canopy inhibited invasion. On the west coast of Vancouver Island in Canada, White and Shurin (2011) [30] found non-linear, density-dependent effects of *Sargassum muticum* on native macroalgal richness. In the present study, the highest concentration of *U. prolifera* in the co-culture system was 3.75 g wet weight L^−1^, and *U. prolifera* at this concentration had no significant effects on *G. lichvoides* (1.25 g wet weight L^−1^). However, the effect of higher co-culture concentrations of *U. prolifera* on *G. lichvoides* should be investigated in the future, as higher concentrations could significantly impact the native species.

Previous studies have reported that the green tide-forming species in the Yellow Sea were *Ulva* (formerly *Enteromorpha*) *linza*–*procera*–*prolifera* complex [40], [41]. *U. prolifera*, the causative species of the world's largest green tide, is distributed widely in the intertidal zones of shores and estuaries around the world because of its tolerance of a wide range of salinity and water temperature, its high growth rate, and its extraordinary capabilities for propagation [8], [42]. In a previous study, we found that the presence of *U. linza* could restrict growth and photosynthesis of *G lemaneiformis*, even when the co-culture density of *U. linza* was equal to that of *G. lemaneiformis* (unpublished data). The present study represents a significant advance in exploring ecological questions about the effects of green tide blooms on the macroalgal community. If hybridization between *U. linza* and *U. prolifera* occurred, a more destructive species could have more serious ecological effects on the marine community.
